# Fast Start-Up Microfluidic Microbial Fuel Cells With Serpentine Microchannel

**DOI:** 10.3389/fmicb.2018.02816

**Published:** 2018-11-20

**Authors:** Xian Luo, Wenyue Xie, Ruijie Wang, Xiaoshuai Wu, Ling Yu, Yan Qiao

**Affiliations:** ^1^Faculty of Materials and Energy, Institute for Clean Energy and Advanced Materials, Southwest University, Chongqing, China; ^2^Chongqing Key Laboratory for Advanced Materials and Technologies of Clean Energies, Chongqing, China; ^3^Chongqing Engineering Research Center for Rapid Diagnosis of Dread Disease, Southwest University, Chongqing, China

**Keywords:** microfluidic microbial fuel cell, interfacial electron transfer, serpentine microchannel, membraneless, laminar flow

## Abstract

Microfluidic microbial fuel cells (MMFCs) are promising green power sources for future ultra-small electronic devices. The MMFCs with co-laminar microfluidic structure are superior to other MMFCs according to their low internal resistance and relative high power density. However, the area for interfacial electron transfer between the bacteria and the anode is quite limited in the typical Y-shaped device, which apparently restricts the current generation performance. In this study, we developed a membraneless MMFC with serpentine microchannel to enhance the interfacial electron transfer and promote the power generation of the device. Owing to the merit of laminar flow, the proposed MMFC was working well without any proton exchange membrane (PEM). At the same time, the serpentine microchannel greatly increased the power density. The S-MMFC catalyzed by *Shewanella putrefaciens* CN32 achieves a peak power density of 360 mW/m^2^ with the optimal channel configuration and the flow rate of 5 ml/h. Meanwhile, this device possesses much shorter start-up time and much longer duration time at high current plateau than the previous reported MMFCs. The presented MMFC appears promising for biochip technology and extends the scope of microfluidic energy.

## Introduction

Microbial fuel cells (MFCs) are bioelectrochemical devices that convert chemical energy of organic substrates to electrical energy via microorganism metabolism (Wang et al., 2011). In recent years, microfluidic MFCs (MMFCs) have been developed for biosensors (Mu et al., 2006; Siu and Chiao, 2008), screening colonies (Li C. et al., 2011; Wang and Su, 2013), or micro power sources (Qian et al., 2009; Choi et al., 2011). Due to the decrease of the characteristic scale in mMFC, the mass transfer of the reactants, and the product is enhanced, which reduces the accumulation of hydrogen ions on the anode side and makes the reaction kinetics stronger. The down-sized MFCs possess high surface area to volume ratio and quick response to reactants (ElMekawy et al., 2013). Typically, an MFC is comprised of an anode chamber and cathode chamber separated by a proton exchange membrane (PEM), which permits H^+^, or other cations, to pass through from the anode chamber to the cathode chamber (Fraiwan et al., 2013). The early MMFCs retain the dual chamber structures with membrane or separator as the macro size devices. However, the power output performances of these down sized devices are quite poor due to the quite high internal resistance (Qian and Morse, 2011). To decrease the internal resistance of the MMFCs, a kind of membrane-less device with co-laminar microfluidic structure has been developed recently (Li Z. et al., 2011; Ye et al., 2013). In this device, a narrow anolyte-catholyte mixing region in the middle of the microchannel replaces the physical separator. The removal of separator between the anode and cathode greatly reduces the internal resistance and meanwhile enhances the power density dramatically.

In 2011, Li et al. firstly reported the laminar-flow based current produced by *Geobacter sulfurreducens* and *Shewanella oneidensis* in a Y-shaped membraneless MMFC with a total volume of 0.3 ml (Li Z. et al., 2011). Currently, the Y-shaped channel is the typical structure in most of the co-laminar MMFCs (Li et al., 2012, 2016; Yoon et al., 2018). In this Y-shaped device, the inlets of anolyte and catholyte are located at the end of the arms (Yang et al., 2016a). The anode and cathode are often placed on the side of the mixing area so that the anode carbon paper is only covered by less than half of the channel. Since the interfacial electron transfer only happens on the fluid channel covered area, the reaction area for these Y-shaped devices is quite low. It is well known that the power generation performance of MFCs is determined by the exoelectrogen adhered on the anode. In this case, the small area for interfacial electron transfer would limit the performance of the Y-shaped co-laminar MMFCs. To improve the performance, Yang et al. designed multiple anode inlets on the Y-shaped channel, which shortened the startup time and also increased the power density by 2 fold (Yang et al., 2016b). It seems that increasing the channel area on the anode could effectively enhance the performance of the co-laminar MMFCs. However, this improved device still needs 40 h lag time before the cell voltage start to increase. It seems that the limited channel area for interfacial electron transfer and biofilm growing on the anode restricts the performance of the device. In this case, appropriate channel design could be a strategy to solve this problem.

In this work, a serpentine micro channel was introduced in the co-laminar MMFCs to increase the interfacial electron transfer on the anode and so far decrease the lag time of the device. The serpentine microchannels have been used in MMFC with polyelectrolyte membrane to maximize the surface to volume ratio in the two-dimensional microfluidic structure (Vigolo et al., 2014). The power generation performance of the serpentine microchannel MFC (S-MMFC) was compared with the Y-shape MMFC and the effect of the channel geometry properties on the current generation was also investigated. The effects of the elongated channel on the lag time, the impedance of the cell as well as the biofilm distribution were also discussed.

## Materials and methods

### Device construction

**Figure 1:**
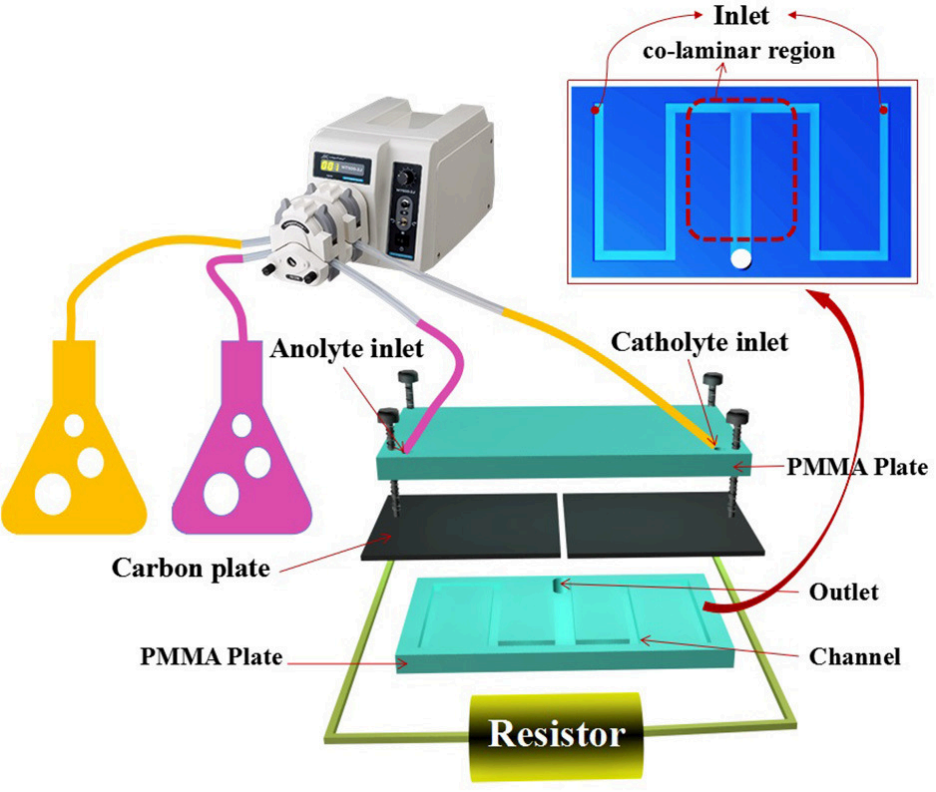
Schematic illustration of S-MMFC construction.

As shown in Figure 1, the standard serpentine microchannel MFC (MMFC, device 1) was consisted of a polymethylmethacrylate (PMMA) plate with channels and a PMMA cover. Two carbon paper electrodes (0.5 mm thick, EDM Supplies Inc., poco grade EDM-3) clamped between them covered the channels. The serpentine channel for anode or cathode included three long segments with 10 mm length for each and two perpendicular short segments with 5 mm length. Both of the width and height of the channel are equal to 1 mm. While, the last long sections for anode and cathode were merged to form a co-laminar region. The whole device was fastened by six M1 screw joints. The liquid enters from anolyte/catholyte inlet of the channel and flows out from the outlet located at the end of the co-laminar region. For device 2, just the part of co-laminar area changed from “T” shape to “Y” shape. For device 3, the channel for each side was extended with one more short segment and one more long segment. In this case, the total channel length for each side was increased from 40 to 55 mm. For device 2, the two short segments were shortened from 5 to 2.5 mm so that the total length for each side was 35 mm. The details about the channel for Y-shaped MMFC and S-MMFC devices were shown in Figure S1.

### Inoculation

A single clone of *Shewanella putrefaciens* CN32 (ATCC, BAA-1097) was grown anaerobically in 5 ml of Luriae Bertani (LB) broth medium (10 g/L sodium chloride,10 g/L tryptone, 5 g/L yeast extract) overnight. 1 ml aliquot of bacterial culture suspension was inoculated in 100 ml of fresh LB medium and incubated with shaking at 30°C until the optical density at 600 nm (OD600) reached about 1.0. The cell pellets were resuspended in M9 buffer (6 g/L Na_2_HPO_4_, 3 g/L KH_2_PO_4_, 0.5 g/L NaCl, 1 g/L NH_4_Cl,1 mM MgSO_4_, 0.1 mM CaCl_2_) containing 18 mm lactate. The anolyte and catholyte were supplied by double pipe peristaltic pump (BT100-2J peristalitic pump, Baoding, China), respectively. The potassium ferricyanide concentration is fixed to 50 mm. The *S. putrefaciens* CN32 in the conical flask was injected into the MFC device by a peristaltic pump. Prior to all experiments, the anolyte was purged with nitrogen gas for 30 min to remove the dissolved oxygen.

### Data acquisition and analysis

For the current generation experiments, an external load with 40 KΩ was connected to the MMFC and the output voltage was recorded with VICTOR 8145B desktop multimeter (Desktop multimeter, Shanghai, China). The current generated by the MMFC operation was monitored as a function of time. By recording the potential drop (V) across the external resistor (R), current in the circuit (I) was calculated via Ohm' s law: *I* = V/R. Output power (P) was calculated via *P* = V × I (Qian et al., 2011). Polarization and power curves were measured by systematically changing the external resistors and recording each V at the equilibrium. Current and power densities were calculated based on the projected anode area (Logan et al., 2006). The polarization and power output curves were measured by varying the output load resistor from 2 to 80 KΩ to monitor the steady-state current, which is measured until a stable reading can be obtained after changing the output load.

Electrochemical impedance spectroscopy (EIS) was conducted on each MMFC to evaluate the anode resistance using an electrochemical workstation (Ye et al., 2013; CHI660E, Shanghai Chenhua, China). During the test, a two-electrode system was employed, which meant anode served as the working electrode and cathode as the counter electrode, respectively. The frequency was varied from 100 to 10 mHz. All tests were performed under closed circuit connecting an external resistance of 40 KΩ between the anode and cathode.

### Biofilm distribution observation

The distribution of the biofilm during the steady-state operation were examined by field emission scanning electron microscope (FESEM) at different magnifications. Before test, the carbon paper anodes were immersed in 4% glutaraldehyde solution overnight and sequentially dehydrated with increasing concentration of ethanol (30, 50, 70, 80, 90, and 100%) for 15 min each step to avoids damaging cell structures during rapid dehydration (Qiao et al., 2015). Prior to FESEM observation, the samples were sputtered with a thin coating layer of platinum.

The overall appearance of the bacterial biofilm on the electrode was observed by the *Bac*light bacterial viability kit. The *Bac*light bacterial viability kit includes mixtures of green fluorescent nucleic acid stain SYTO 9 and red fluorescent nucleic acid stain propidium iodide (PI). The process is as following: (1) Centrifuge the vial before opening; (2) Dilute 10^*^Assay Buffer to 1^*^Assay Buffer with distilled water; (3) Wash electrode twice with phosphate buffer saline(PBS) and re-suspend electrode in desired volume of 1^*^Assay Buffer. (4) Add 5 μl PI Staining solution to 95 μl electrodel and incubate it for 5~30 min. (5) Apply fluorescence microscopy.

## Results

### Performance of S-MMFC vs. Y-MMFC

**Figure 2:**
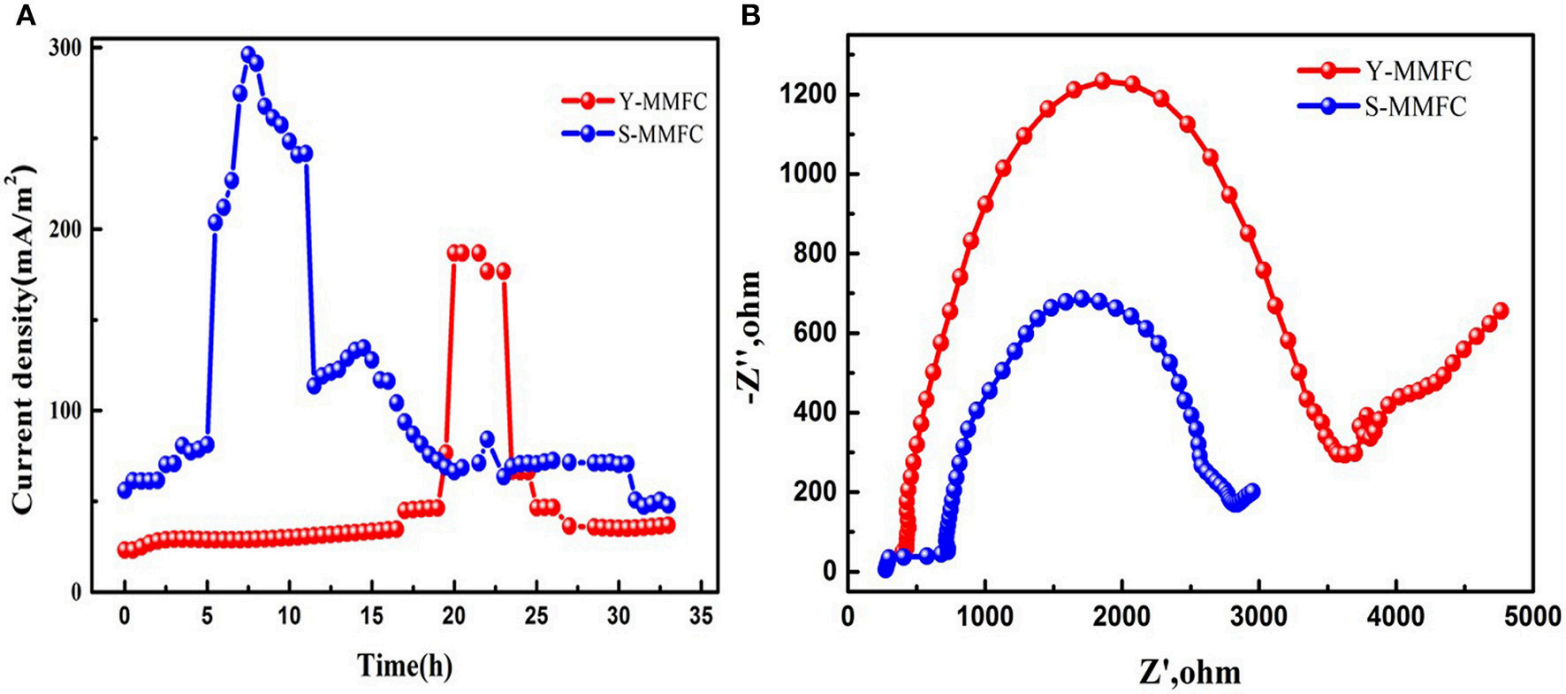
Comparison of S-MMFC and Y-MMFC. **(A)** current generation profiles. **(B)** Nyquist Plots.

A contrastive analysis of Y-shape and S-shape MMFCs was conducted. The current generation profiles (Figure 2A) show that the S-MMFC possesses faster start-up process, which only takes 6 h to reach the maximum current density. While the lag time for Y-MMFC lasts around 20 h and the maximum current density is much lower than that of S-MMFC. The Nyquist plots of electrochemical impedance spectra analysis suggest that the S-MMFC has smaller internal resistance than that of Y-MMFC (Figure 2B). Considering the co-laminar area for these two devices are almost same, the differences on the current generation and internal resistance could be due to the diverse designs of electrode area including the geometry properties.

### Discharging curves of S-MMFCS with different channel configuration

**Figure 3:**
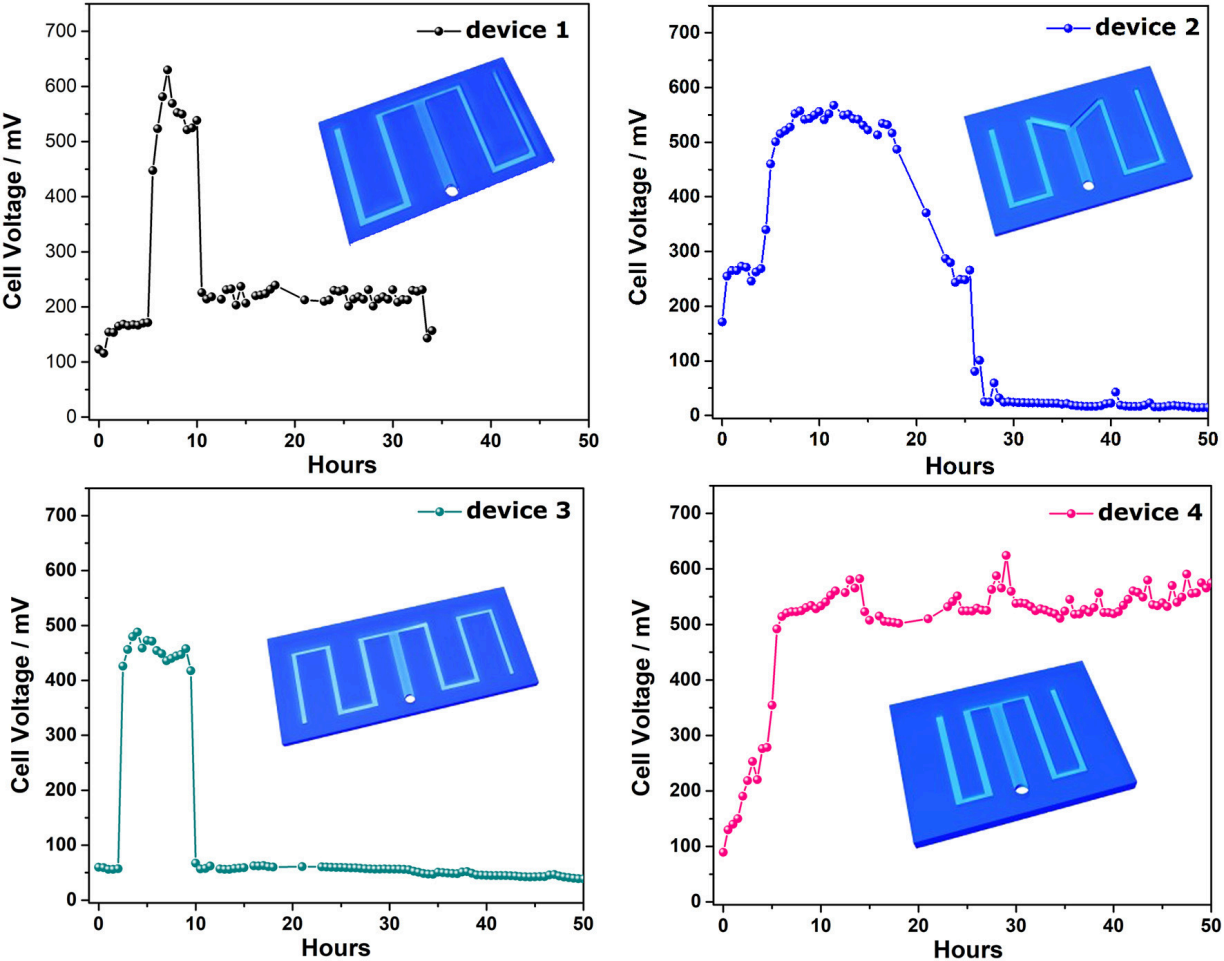
Current generation profiles of different designs of S-MMFCs.

To investigate the effect of the channel geometry on the current generation profile of the S-MMFC, Four devices with different channel configurations were developed as shown in Figure 3. For device 1 and device 2, the total area of the micro channel is almost same and the carbon paper electrodes for these two devices are same. It is interesting that the lag time of these two devices are similar (5 h) but the maximum cell voltage of device 1 is a little bit higher than that of device 2. It is also noted that the duration time at high voltage plateau for device 2 is much longer. The reason might be that the sharp angle in the channel changes the wall shear stresses and also the distribution of bacteria cells as well as the protons in the channel. For device 3, the increased channel length seems leading to a shorter lag time (2 h) but the maximum value is lower than device 1. According to the calculated hydraulic retention time (HRT, Table S1), the elongated channel will increase the HRT and thus facilitate the adhesion of bacteria. However, the long channel will also result in a huge proton transfer resistance. For device 4, the shortened channel and the decreased electrode area delivers much longer duration at high voltage plateau and no obvious lag period can be found. Although the HRT of device 4 is lower than other three devices, the shortened and shrinked channel configuration will decrease the internal resistance of the device. Therefore, the lag phase is not observed on the discharging curve and the duration time on maximum cell voltage is much longer than other devices (Figure S2). The drop-off of the cell voltage could be due to the consumption of the substrate in the channel since the voltage can be recovered when fresh lactate medium flow through the device (data not shown).

### Power curves and electrochemical impedance spectra

**Figure 4:**
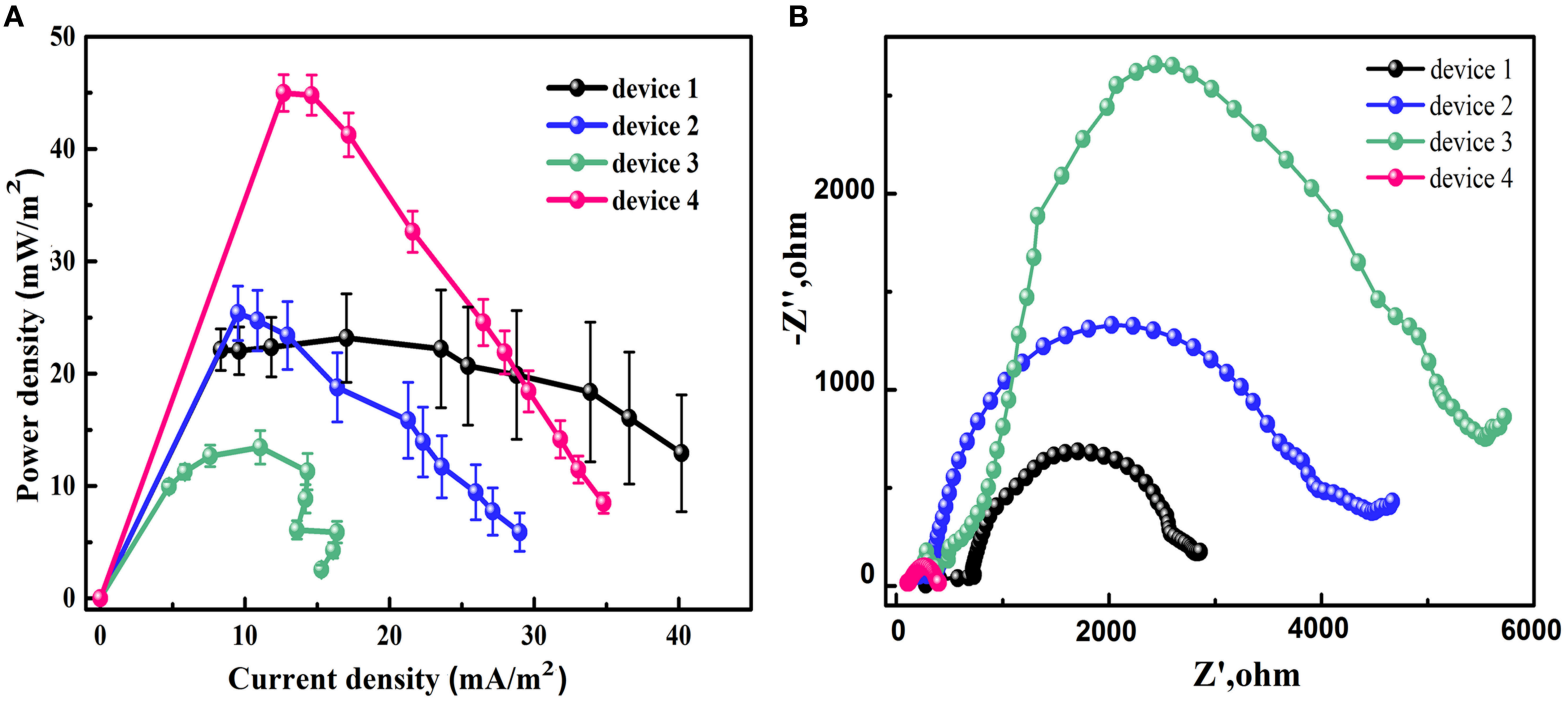
Power curves **(A)** and Nyquist plots **(B)** of different designs of S-MMFCs.

To understand the reason of the effect of the channel design on the cell performance, the power curves and the electrochemical impedance spectra of different devices were examined. The power density was calculated on the anode channel area, which were 40, 41, 55, and 35 mm^2^ for device 1, 2, 3, and 4, respectively. In Figure 4A, the power curves of different devices show that the device 4 has the highest maximum power density since it has lowest anode area. The maximum power density for device 1 and device 2 are almost same while the device 3 has the lowest maximum power density. This result is in agreement with the cell voltage data. From Figure 4B, the device 4 possesses the lowest impedance while the device 3 has highest one. This result is in accordance with the previous discussion that the shorter distance between the channel input end and the ion exchange area will decrease the internal resistance of the MMFC. It is also noted that the impedance of device 1 is smaller than that of device 2 although the channel length and the electrode area for them are almost same. The reason might be that the device 2 possesses larger proton transfer resistance than device 1. The detailed mechanism needs further investigation on the microfluidic dynamitic process.

### Biofilm distribution observation

**Figure 5:**
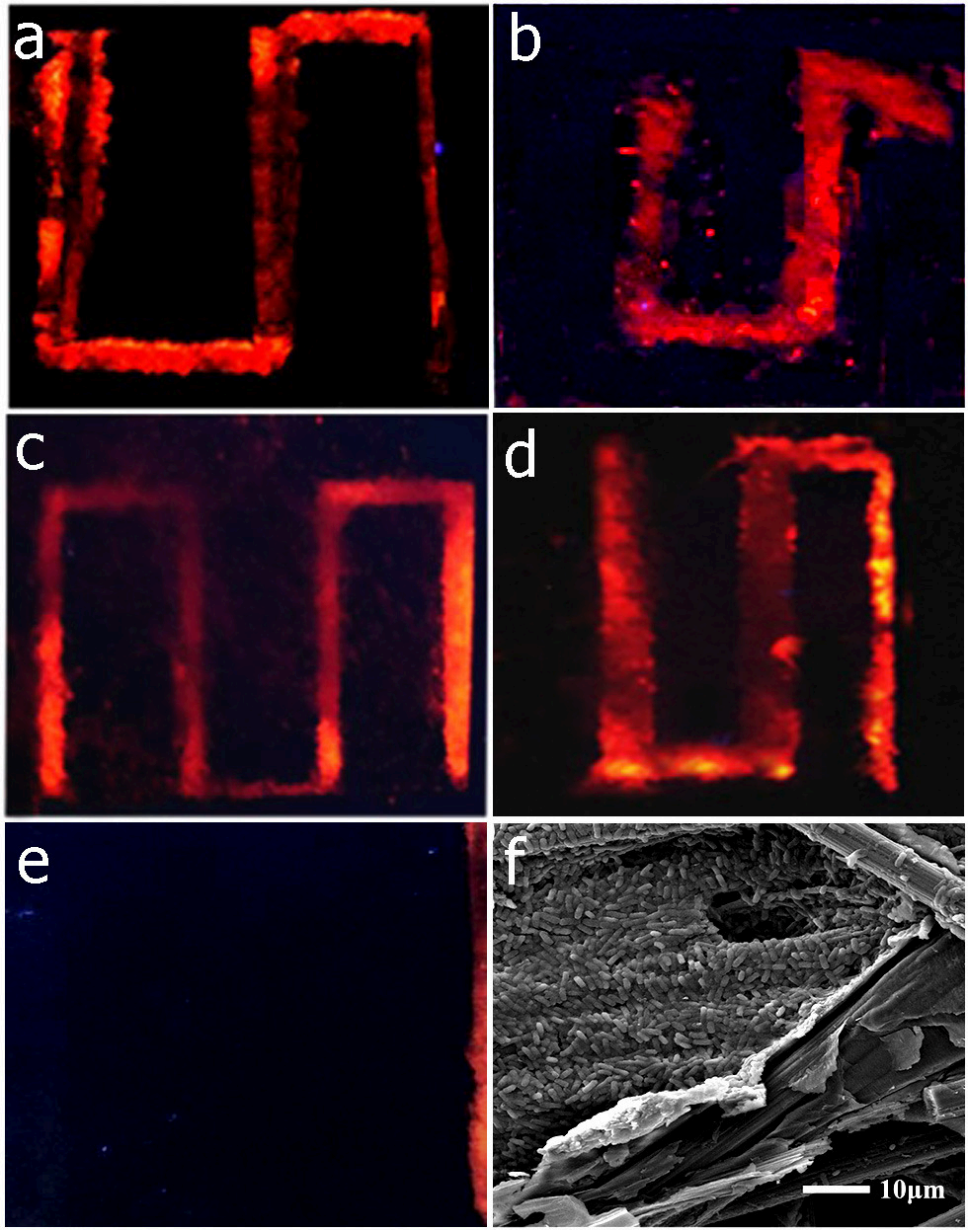
Fluorescence photographs **(a-e)** of carbon paper anodes of different devices to show the biofilm distribution (**a**: device 1, **b**: device 2, **c**: device 3, **d**: device 4, **e**: Y-shaped device) and SEM micrograph of the boundary of the channel in device 1 **(f)**.

To clarify the biofilm distribution of different MMFCs, the anode carbon paper of each device was dyed with pyridine iodide (PI) after discharge. Figure 5 shows the biofilm distribution of each device. The shape of the biofilm on the carbon paper is same as the channel design, which suggests that biofilm only grows at the place with electrolyte. The cell density is higher at the segment near the co-laminar region as the flowrate is slower at this place. From this results, it is obvious that the S-MMFCs possess much more biofilm loading amount than that of Y-MMFC so that they have much shorter lag time that that of Y-MMFC. The Figure 5e shows the morphology of carbon paper electrode at channel boundary (device 1). A dense biofilm can be observed with a clearly separation between the biofilm loaded surface and the original surface of the carbon paper. To investigate the time dependent biofilm growth profile, the morphologies of the carbon paper anode of device 4 at different time were observed with FESEM (Figure S3). The results show that the cell density is increased as the discharging time and a dense biofilm could be observed after 24 h.

### Cell performance comparison

The cell performance of the S-MMFC device 4 was also compared with the reported μMFCs with *Shewanella* as biocatalysts. According to the data listed in Table 1, the S-MMFC developed in this work not only has shortest lag time but also delivers much higher maximum power density. The S-MMFC catalyzed by *S. putrefaciens* CN32 achieves a peak power density of 360 mW/m^2^ with the optimal channel shape at the flow rate of 5 ml /h. Meanwhile, this device possesses much longer duration time at high current plateau than the previous reported MMFCs.

**Table 1:** Cell performance comparison between reported MMFCs.

**Anode**	**Cathode**	**Anode volume (cm^3^)**	**Inoculum**	**Membrane**	**Lag time**	**I _max_ (mA/m^2^)**	**P _max_ (mW/m^2^)**	**References**
Au	Au	0.014	*Shewanella oneidensis* MR-1	N/A	36 h	254.2	N/A	Li Z. et al., 2011
Gold	Carbon cloth	0.15	*Shewanella oneidensis* MR-1	N/A	N/A	130	1.5	Qian et al., 2011
PCL microfiber	Au	0.28	*Shewanella oneidensis* MR-1	Nafion 117	N/A	28.5	6.5	Fraiwan et al., 2013
Carbon paper	Carbon paper	0.35	*Shewanella putrefaciens* CN32	N/A	5 h	300	46	This work

## Discussion

From the above results, it is noted that the elongated microchannel with higher HRT would significantly accelerate the start-up process of the MMFC but also dramatically increase the internal resistance at the same time. It is believed that the interfacial electron transfer between the anode respiring bacteria and the electrode is one of the most important steps for MFC power generation. The accelerated colonization of anode respiring bacteria could significantly shorten the start-up time (Wang et al., 2009; Boghani et al., 2013). For the microfluidic MFC devices under continuous flow, this electron transfer process only takes place in the microchannels. In this case, the elongated microchannels would provide larger reaction area for interfacial electron transfer, which might be the reason for the fast start-up of these S-MMFCs.

On the other hand, the elongated microchannels with expended electrode area would result in the increase of the internal resistance due to the large proton transfer resistance. To achieve better power generation performance, the shrunk microchannel design with long channel length and small electrode area could be a good solution. In addition, the wall shear stress of the channel should also be considered for the channel design.

## Author contributions

XL and YQ designed the experiments and wrote the manuscript. XL was responsible for performing all experiments, WX assisted with the fluorescence observation, RW and XW assisted with the FESEM observation, LY assisted with the device fabrication. All authors contributed to interpreting the results, critically revising the manuscript for important intellectual content, and approving the final manuscript.

### Conflict of interest statement

The authors declare that the research was conducted in the absence of any commercial or financial relationships that could be construed as a potential conflict of interest.
