# Supplementary material for: Fast Start-Up Microfluidic Microbial Fuel Cells With Serpentine Microchannel
Source: Front Microbiol. 2018 Nov 20;9:2816. doi: 10.3389/fmicb.2018.02816 (PMC6256063; doi:10.3389/fmicb.2018.02816)
Supplement: Supplementary file 1 [file Data_Sheet_1.pdf]

Supporting information

Fast start-up microfluidic microbial fuel cells with serpentine microchannel

Xian Luo, Wenyue Xie, Ruijie Wang, Xiaoshuai Wu, Ling Yu, Yan Qiao*

a. Institute for Clean Energy and Advanced Materials, Faculty of Materials and Energy, Southwest University, Chongqing 400715, China

b. Chongqing Key Laboratory for Advanced Materials and Technologies of Clean Energies, Chongqing 400715, P.R. China

c. Chongqing Engineering Research Center for Rapid Diagnosis of Dread Disease, Southwest University, Chongqing 400715, China

*Corresponding author. Tel/Fax: +86-023-68254842; E-mail: yanqiao@swu.edu.cn

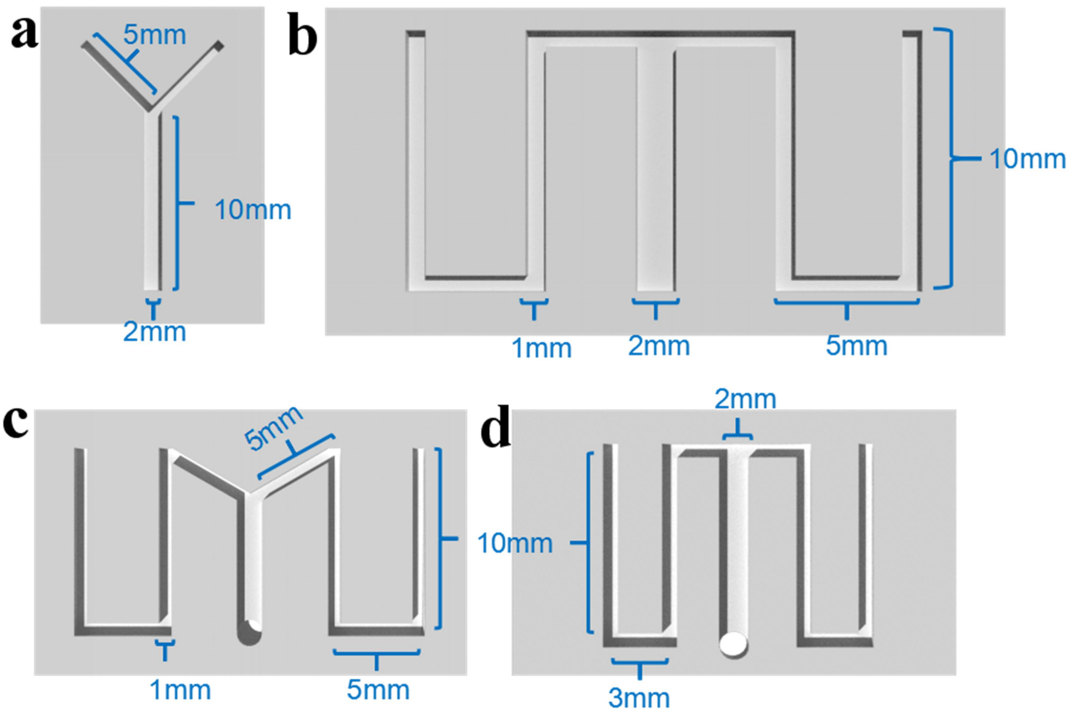

Fig. S1 Details about the channel configurations of Y-type MMFC (a), S-type MMFC device 1 (b), S-type MMFC device 2 (c) and S-type MMFC device 4 (d).

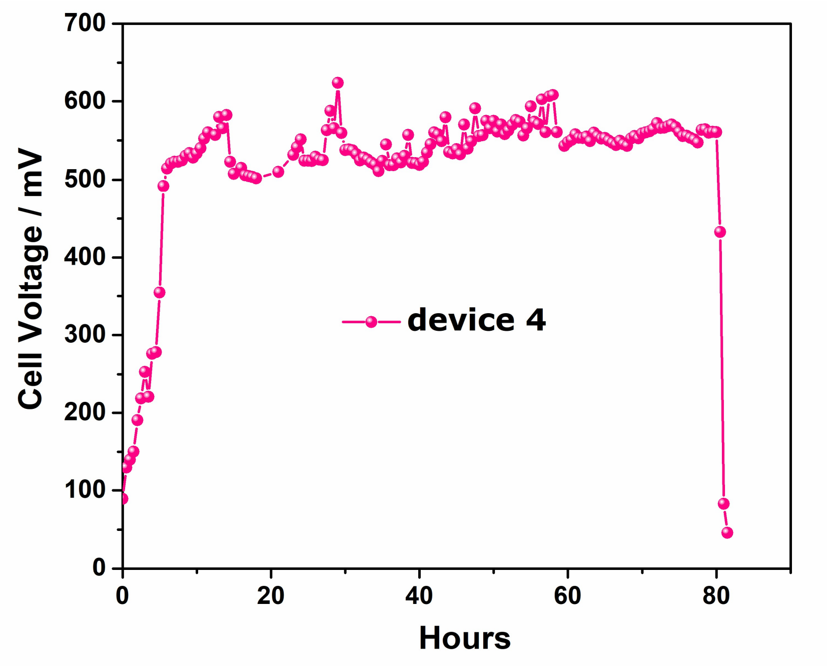

Fig. S2 Discharging curve of S-type MMFC device 4 for long time operation.

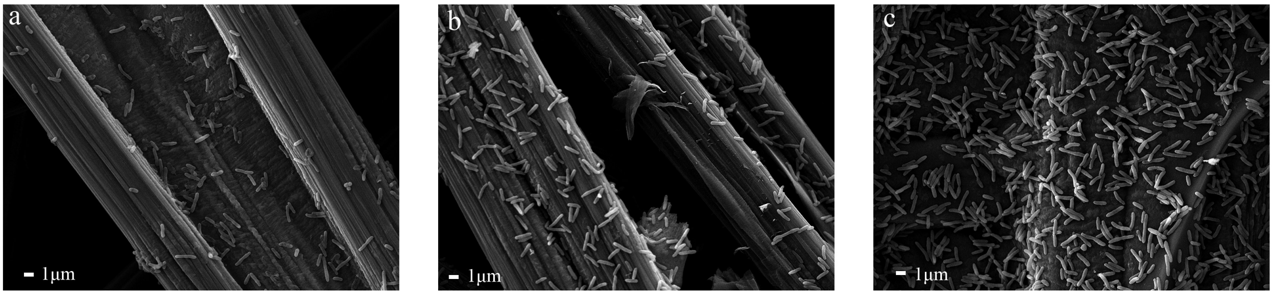

Fig. S3 Growth of bacterial biofilm on the electrode of device 4 at different time. (a, 6 hours; b, 12 hours; c, 24 hours)

Calculation of hydraulic retention time (HRT)

Since the laminar flow zone of the four devices is the same in velocity, density, viscosity and pipe diameter, when the flow rate is 5ml/h. $Re= du\rho/\mu$, $Re=1.3$

Table S1. According to the formula HRT = V / Q (El Hamouri *et al.*, 2007; Najafpour *et al.*, 2006), the HRT of different devices is calculated.

Type	V	Q	HRT
Device1	$8*10^{-10}m^3$	$5*10^{-7}m^3$	$1.6*10^{-3}$
Device2	$8*10^{-10}m^3$	$5*10^{-7}m^3$	$1.6*10^{-3}$
Device3	$11*10^{-10}m^3$	$5*10^{-7}m^3$	$2.2*10^{-3}$
Device4	$7.2*10^{-10}m^3$	$5*10^{-7}m^3$	$1.44*10^{-3}$

References

El Hamouri, B., Nazih, J., and Lahjouj, J. (2007), Subsurface-horizontal flow constructed wetland for sewage treatment under Moroccan climate conditions. Desalination 215 153-158, doi:https://doi.org/10.1016/j.desal.2006.11.018.

Najafpour, G.D., Zinatizadeh, A.A.L., Mohamed, A.R., Hasnain Isa, M., and Nasrollahzadeh, H. (2006), High-rate anaerobic digestion of palm oil mill effluent in an upflow anaerobic sludge-fixed film bioreactor. Process Biochem 41 370-379, doi:https://doi.org/10.1016/j.procbio.2005.06.031.
